# Integration of Novel Sensors and Machine Learning for Predictive Maintenance in Medium Voltage Switchgear to Enable the Energy and Mobility Revolutions

**DOI:** 10.3390/s20072099

**Published:** 2020-04-08

**Authors:** Martin W. Hoffmann, Stephan Wildermuth, Ralf Gitzel, Aydin Boyaci, Jörg Gebhardt, Holger Kaul, Ido Amihai, Bodo Forg, Michael Suriyah, Thomas Leibfried, Volker Stich, Jan Hicking, Martin Bremer, Lars Kaminski, Daniel Beverungen, Philipp zur Heiden, Tanja Tornede

**Affiliations:** 1 ABB AG, Corporate Research Germany, 68526 Ladenburg, Germany; 2 Heimann Sensor GmbH, 01109 Dresden, Germany; 3 Institute of Electric Energy Systems and High Voltage Technology, Karlsruhe Institute of Technology (KIT), 76131 Karlsruhe, Germany; 4 FIR (Institute for Industrial Management) at the RWTH Aachen University, 52074Aachen, Germany; 5 Chair of Business Information Systems, Paderborn University, 33098 Paderborn, Germany; 6 Software Innovation Campus Paderborn, Department of Computer Science and Heinz Nixdorf Institute, Paderborn University, 33098 Paderborn, Germany

**Keywords:** energy revolution, condition monitoring, switchgear, infrared sensor, predictive maintenance, machine learning, thermal monitoring, business model

## Abstract

The development of renewable energies and smart mobility has profoundly impacted the future of the distribution grid. An increasing bidirectional energy flow stresses the assets of the distribution grid, especially medium voltage switchgear. This calls for improved maintenance strategies to prevent critical failures. Predictive maintenance, a maintenance strategy relying on current condition data of assets, serves as a guideline. Novel sensors covering thermal, mechanical, and partial discharge aspects of switchgear, enable continuous condition monitoring of some of the most critical assets of the distribution grid. Combined with machine learning algorithms, the demands put on the distribution grid by the energy and mobility revolutions can be handled. In this paper, we review the current state-of-the-art of all aspects of condition monitoring for medium voltage switchgear. Furthermore, we present an approach to develop a predictive maintenance system based on novel sensors and machine learning. We show how the existing medium voltage grid infrastructure can adapt these new needs on an economic scale.

## 1. Introduction

Germany’s energy policy requires the electricity system to be more efficient, environmentally friendly, and a source of affordable energy for everyone [1,2]. At the same time, the upcoming mobility revolution has a significant impact on the use of the grid. As a result, there will be substantial changes to our distribution grid system’s characteristics. On the generation side, renewable energy will eventually replace a large portion of current conventional energy production from fossil fuels. Therefore, we face a decentralization of the creation of energy. Centralized fossil fuel power plants provided stable and easily controllable energy production. In contrast, many renewables, such as wind and solar, can destabilize the production through their unplannable production patterns that are highly weather-dependent [3,4]. Both production overloads and gaps will occur. On the demand side, electric mobility will result in higher consumption peaks and a higher demand for energy. This is caused by coupling mobility to the power sector and the specific consumption patterns of mobility applications. The combination of these developments leads to a major challenge for the distribution grid.

To accomplish this challenge, system operators must renew the assets in their grids to increase the system’s flexibility [5,6]. Although Germany’s grid is currently one of the most stable grids in the world, some of its essential components will have to be replaced. There is an unusually high renovation demand on the medium voltage range because increased electric mobility and decentralized electricity production mainly affect switching patterns and peak performances within the medium voltage range [7,8]. We must put in place an efficient monitoring system for both the existing and the new components to avoid disturbances and failures.

**Figure 1:**
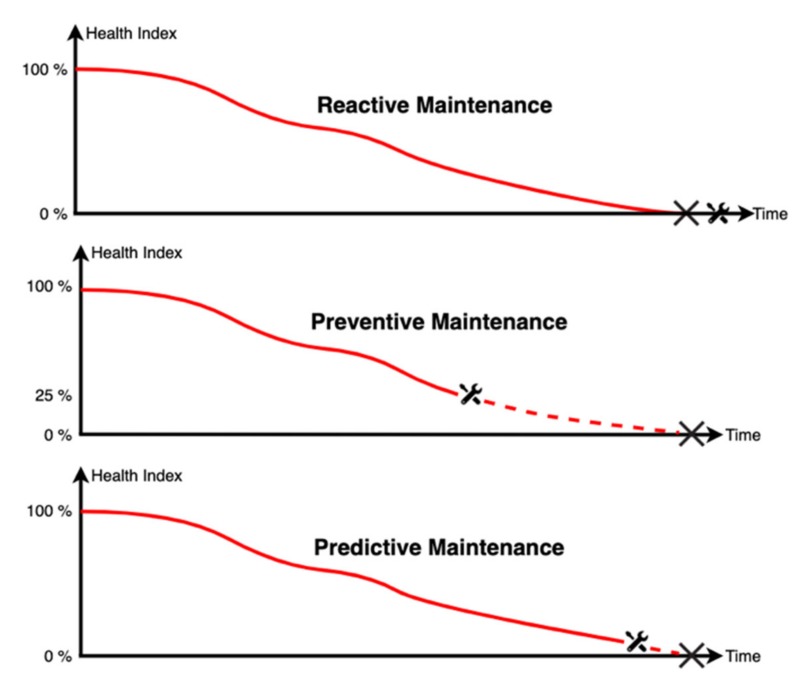
Example sketch of the maintenance strategies: reactive maintenance, where maintenance is applied after a failure occurred; preventive maintenance, where maintenance is always applied when the health index reaches 25%; predictive maintenance, where maintenance is done directly before the failure occurs.

To achieve the necessary grid stability, grid operators must improve monitoring and asset communication so that network operations can be as flexible as possible. Because of the high occurrences and irregularities of switching events, the essential components for power grids, e.g., switchgear (Section 2.1) and circuit breakers, cannot be maintained at fixed intervals anymore. In general, there are three strategies for maintaining industrial equipment: reactive, preventive, and predictive maintenance [9], visualized in Figure 1. When applying reactive maintenance, no action is taken until machines or equipment fail. In this case, the full lifetime of the system is exploited, but severe failures can occur unexpectedly, which will result in expensive maintenance and potentially dangerous situations.

Preventive maintenance is triggered based on statistics, e.g., hours of operation, or elapsed time since the last maintenance, leading to periodic maintenance processes, where the actual conditions of the equipment are not considered. Usually, there is some healthy lifetime left at the points of maintenance, and there is a risk of over-maintenance, e.g., excessive lubrification of moving parts.

Predictive maintenance combines condition monitoring, system efficiency, and other indicators to identify failures or loss of efficiency in the future. Maintenance is scheduled based on the monitored status of the equipment so that changes in its condition may trigger corrective actions. This exploits the lifetime of the system to a maximum without failing. Predictive maintenance combines cost-, work-, and environmental efficiency, making it the desired maintenance strategy to use on the supply grid. Unlike preventive maintenance, predictive approaches require intelligent algorithms to determine the most effective and risk-free maintenance plan. These algorithms can be based on simulations or AI methods such as expert systems or machine learning [10]. In fact, predictive maintenance is currently the most common application of AI in the industrial sector [11,12].

In medium voltage switchgears, three main challenges can be pointed out by applying predictive maintenance concepts [13]. The first difficulty is to find suitable sensors that are capable to measure the critical physical quantities in a reliable and a robust way over the lifetime of the switchgear. Additionally, the sensors must withstand extreme environmental conditions under which switchgears are operated all over the world. A further challenge is given by the lack of measurement data. For temperature monitoring, continuous measurements are rare or even not existent for the switchgear over its long lifetime. Regarding breaker drive monitoring, switching operations are only performed few times a year mostly for maintenance purposes. Therefore, measurement data is only rarely available for both use-cases which builds the fundament for the development of AI/ML algorithms. In breaker drive monitoring, the situation is further exacerbated by the fact that the duration of a switching operation is extremely short in the range of tens of millisecond. Thus, the interpretation of the measured data and the development of reliable prediction algorithms is very challenging. 

**Figure 2:**
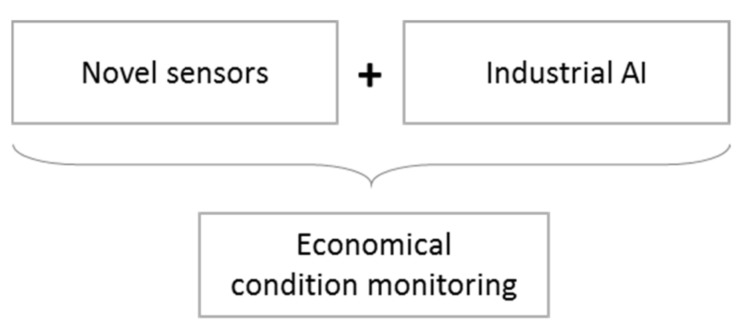
Novel sensors combined with industrial artificial intelligence methods can lead to more economical condition monitoring solutions.

To create an optimized computerized maintenance management system (CMMS) [14] for predictive maintenance in the distribution grid, grid operators must provide and analyze the right data. This aligns with the interest of operators to increase awareness in predictive maintenance and condition monitoring [13,15]. For data acquisition, remote terminal units (RTU) and sensor technologies are essential. As an example, temperature monitoring via infrared can provide data for early failure detection cost-efficiently [16]. For the data analysis, a defragmented infrastructure for big data analysis and the use of artificial intelligence (AI) methods [17] help to simplify and initiate decision support from complex industrial data sets [18,19,20]. Combining methods of industrial AI with novel sensing technology enables new economical, technical solutions, such as condition monitoring and predictive maintenance (Figure 2). Furthermore, the combination of the data analysis with geographic information systems (GIS) accelerates the maintenance process [21].

Within this work, we present an industrial use case, based on the current and future situation of Germany’s power distribution grid. This paper builds upon these changes and the resulting challenges, also addressed in the FLEMING (https://www.projekt-fleming.de) research project. The focus lies on efficient predictive maintenance for the essential components of the grid’s medium voltage range. 

**Figure 3:**
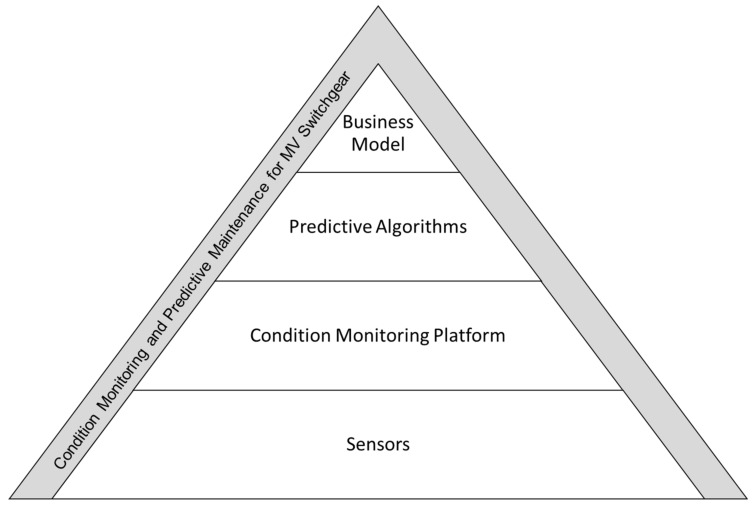
A comprehensive view of condition monitoring and predictive maintenance of medium voltage switchgear.

In the following, we describe our approach to predictive maintenance of medium voltage switchgear systems. The reasoning is illustrated in Figure 3. Existing and novel sensors build the technical foundations for this approach. The signals generated in these sensors are processed in condition monitoring platforms, e.g., in the form of mechanical systems attached to switchgear systems. Using the platform, we determine the current condition state of the different parts of the switchgear. Maintenance and operations actions can be derived from the condition. Predictive algorithms utilize machine learning methods and process the data from the condition monitoring platform. For this purpose, the data can be linked to further data sources, e.g., other switchgear or sensors, to predict changes in asset condition in the future. This enables the planning of an improved maintenance strategy for the individual switchgear. For industrial players to adopt such technological advances, a suitable and scalable business model for condition monitoring and predictive maintenance of medium voltage switchgear needs to be developed and tested.

The proposed approach corresponds with more holistic concepts like the digital twin and cyber-physical systems (CPS) [22]. These concepts cover a broader range of the industrial asset life-cycle, i.e., from engineering and commissioning to operations and maintenance. Applications within CPS, so-called smart services [23], therefore range from integrated engineering tools, over production optimizations to predictive and prescriptive maintenance. The basis for these smart services is multi-model data from a wide variety of computer systems used in the different life-cycle phases by the asset manufacturers, integrators and operators [24]. The data are to be collected in an industrial internet of things (IIoT) fashion and form a digital twin of the asset in the virtual, or cyber, world. Originally, the concept of digital twins focusses on the engineering phase of the asset life-cycle in production systems [25], but more and more extends to all product life-cycle phases [26].

In the present use-case, the medium-voltage switchgear is the physical asset, the condition data can be considered its digital twin in the virtual world, and the predictive maintenance applications are the smart service. The use-case is currently limited to the operations and maintenance life-cycle phase but may utilize data from other phases in the development of the smart service, i.e., simulation know-how of the switchgear for the development of machine learning applications. This is mostly due to the brownfield market, and thus retrofit solutions are to be favored over novel systems addressing greenfield installations.

This review intends to combine a survey of real-world industrial problems with an overview of technological state-of-the-art. Some remarkable challenges of energy and mobility revolutions are elaborated, and promising solutions, e.g., regarding the future of switchgear operations, are discussed. These solution proposals are planned to be investigated in the near future to enable transformations in the energy sector, which have been identified as of public interest.

The structure of the paper is as follows: First, we introduce switchgear function and its components, as well as failure modes and monitoring approaches for each switchgear component (Section 2). We then describe the sensors technology required for such monitoring approaches (Section 3), followed by an overview of the state-of-the-art machine learning methods for predictive maintenance as well as a motivation for using machine learning instead of alternative approaches (Section 4). In Section 5, a service-based business approach is detailed, which can leverage recent technological developments. In the end, we discuss our findings.

## 2. Technology: Distribution Grid Assets and Monitoring

**Figure 4:**
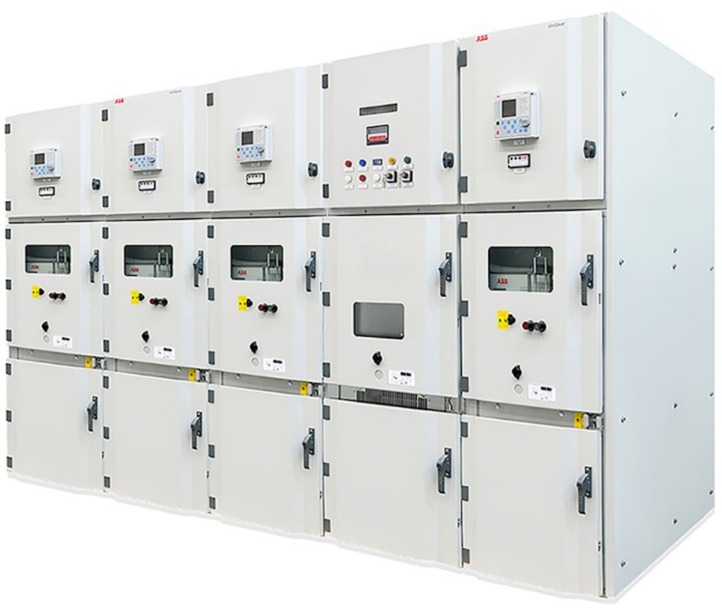
Medium voltage switchgear with five panels.

Switchgear is an essential element in an electrical grid that has both protective and control roles. With switchgear, it is possible to interrupt an electrical circuit, e.g., to prevent further damage after a fault or to modify parts of the circuit. There are many different types of switchgear. In this paper, we focus on medium voltage switchgear (Figure 4) and its key component, the circuit breaker.

### 2.1. Medium-Voltage Switchgear 

**Figure 5:**
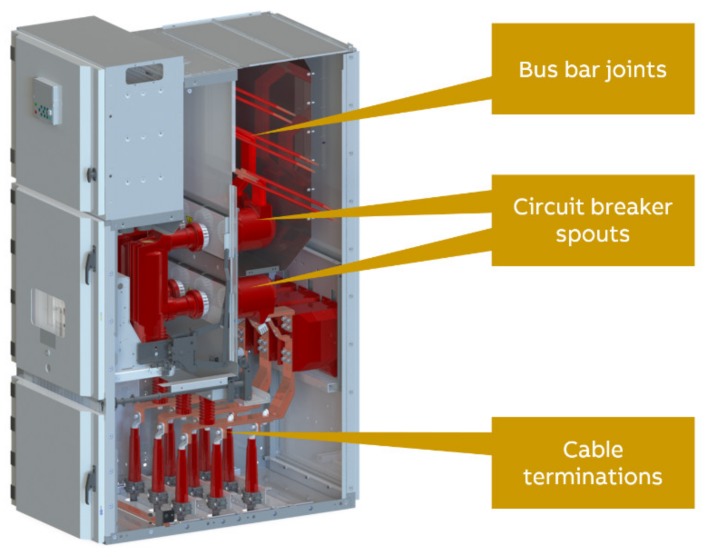
A medium voltage switchgear panel.

Medium voltage switchgear deployed inside closed buildings is a so-called line-up consisting of typically tens of switchgear panels (Figure 4). Often, air is used as an insulation medium, allowing for greater flexibility in designing and extending the line-up. The main functionality and requirements of a medium voltage switchgear panel are the following: Segregation of electrical failures, e.g., arc flash, inside of one switchgear, guarantee safe operation by persons, serviceability & compactness, ability to disconnect and ground parts of the switchgear, long-time operation for several decades and limitation of heat-up of current-carrying parts. These aspects mainly dictate the fundamental design of a modern medium voltage switchgear. The entire electrical system is metal-enclosed, with doors often supervised by interlock systems. The switchgear is protected towards its neighboring switchgear by segregation walls and may be equipped with an air blast duct to guide away hot gas from an arc flash via a chimney integrated into the switchgear. Typically, switchgear is furthermore divided into several compartments: cable, breaker, and bus bar compartment for the high voltage carrying components (e.g., current-carrying, opening, and closing, insulation) as well as a compartment for the low-voltage control equipment (Figure 5). The leading protection equipment, e.g., circuit breakers, can be removed from the switchgear by a sliding mechanism allowing to take out the breakers for service or replacement. Additional functionality, e.g., current and voltage transformers and sensors, are integrated into the switchgear. For safe service operation and reconfiguration, the switchgear is typically equipped with an earthing switch.

Generally, the medium voltage line-up consists of a central bus bar system running through all panels of the line-up. The central system consists of three horizontal individual bus bars, one for each phase. Inside each panel, vertical feeder bus bars are connected to the central system to connect to the components in the individual panel electrically. The individual panels can be configured as an incomer, feeder, bus-coupler, etc. A large variety of panel topologies can be found in the field as its detailed geometry highly depends on rated voltage (7.2 kV–36 kV) and current ratings (630–3150 A).

### 2.2. Breaker Drive Monitoring 

**Figure 6:**
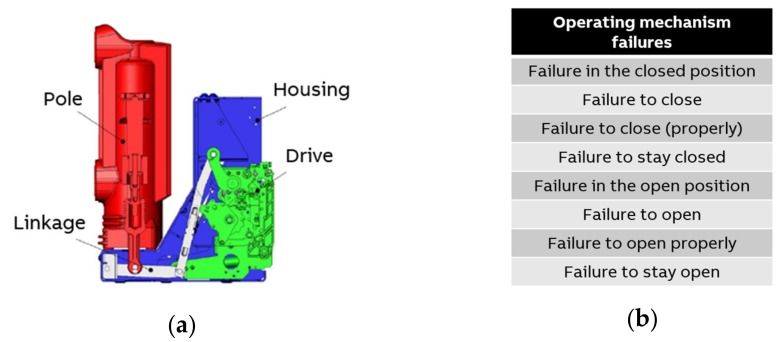
(**a**) Typical medium voltage circuit breaker (CAD model); (**b**) Operating mechanism failures of circuit breakers according to [29].

In switchgear, the critical task of a circuit breaker is to protect the electrical current from damage by interrupting fault currents and isolating faulty parts from the power grid. From a mechanical point of view, the circuit breaker is often grouped into four subsystems: drive, linkage, pole, and housing (Figure 6a). Spring-driven mechanisms are widely used in most of the applications where the drive subsystem provides the energy for closing and opening operations. The linkage represents the transmission mechanism between the drive and the pole that contains the electrical contacts interrupting the fault currents. The metal housing surrounds the drive and the linkage where the pole is enclosed with unique insulating material. 

A German study of failure data of electrical components in the medium voltage distribution grid [27,28] reveals that the circuit breaker is the main component prone to failure in medium voltage switchgear. About 90% of all circuit breaker failures are mechanical [27,28] and therefore occur in the operating mechanism [29] and the breaker drive, respectively (Figure 6b). 

The IEEE guideline [30] gives a generic overview of several failure modes that may occur for circuit breakers in general. Each failure mode is described in detail with possible causes, effects, and characteristics, as well as offering monitoring options. Based on [30,31], the authors in [13] identify the most critical failure modes for today’s medium voltage circuit breakers, also with a focus on the breaker drive. Several methods can be concluded from the state-of-the-art for monitoring the breaker drive. One method is to evaluate the contact travel time of closing and opening operation, which indicates the need for maintenance of the breaker drive [32]. The detailed measurement of the contact travel gives further insight into the breaker health status [33]. A further common method is to analyze the vibration signals at one location of the circuit breaker during closing and opening operations [34,35]. Mechanical anomalies can be detected by comparing it with a healthy state. Signal processing methods like short-time FFTs and Wavelet analyses can support failure detection [36].

However, the review of the technical maturity of monitoring options [13] concludes that the condition monitoring and diagnostics of the breaker drive still represents an open research topic. This is because the kinematic chain to the poles, representing the breaker drive, is very complex and consists of many mechanical parts (joints/bearings, springs, dampers, lever arms, sheet metal, rubber stops, electrical contacts, etc.) which may potentially fail.

### 2.3. Thermal Monitoring 

**Figure 7:**
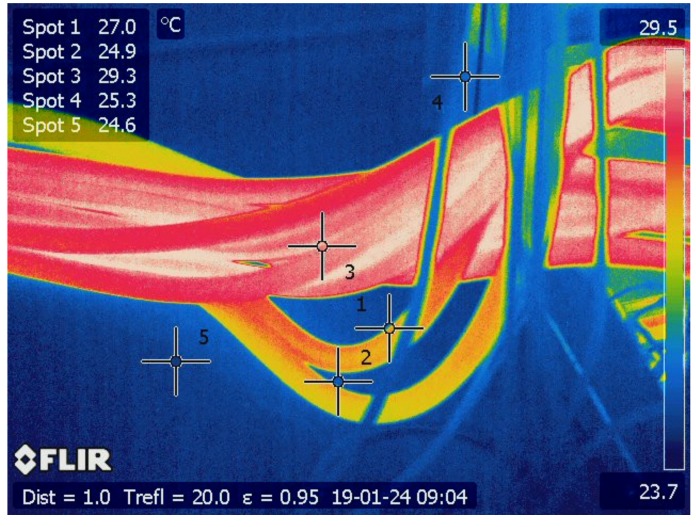
Current in cables visible due to increased heat.

The passage of electric current through a conductor generates heat in a process called Joule heating (Figure 7). According to Joule’s first law, both the current as well as the resistance influence the amount of heat generated: $P\propto I^{2}R$. Since many faults (e.g., deterioration, lose connections, or corrosion) increase the resistance of electrical contacts, their presence can be detected via temperature monitoring (e.g., [16,37,38,39]). Moreover, an increased current will also produce more heat, which can speed up deterioration and reduce the life expectancy of electrical equipment [16].

In switchgear, several electrical connections are established by screwing together metal conductors such as the busbars. These connections can become loose due to ambient vibration, e.g., if mounted on nautical vessels or next to heavy-duty production facilities. Another common cause of looseness is the attachment of the screws at the wrong torque, e.g., after a maintenance operation. 

### 2.4. Partial Discharge Monitoring

According to IEC standard 60,270 [40], partial discharge (PD) is a localized dielectric breakdown of a small portion of an electrical insulation system under high voltage stress, which partially bridges the gap between two conductors which are put on different electrical potential. PD is generally divided into two major sub-groups, internal and external PD, depending on their occurrence [41].

These discharges indicate that locally the electric insulation cannot withstand the electric field stress applied to them. While the correlation of PD occurrence with a subsequent imminent breakdown of an electric system is often not clear, PDs are known to be visible in a large number of cases, where a breakdown occurred later. PDs usually have small magnitude, but over time they can cause progressive deterioration of insulation. The electrical insulation subjected to high electrical fields starts to degrade due to mechanical, thermal, and electrical stress. PD is both symptomatic of insulation breakdown and a mechanism for further insulation damage. Therefore, the detection of PD strength and type can be used to evaluate the instantaneous condition of the insulation. Furthermore, its degradation over time (see, e.g., [42] and references therein) may be predicted based on sensing a gradual increase in PD activity. Sensing concepts for PD detection are briefly described in Section 3.3. 

A specific field of recent interest [43] which will be addressed by studies in this project, is to gather data to be able to derive correlations between PD activity and power quality. The ongoing trend towards renewables and electrical mobility, both of which are inherently coupled with an increase in the use of semiconductor-based switching power converters, changes the electrical stress that the insulation system must undergo. While previously, the voltage on an AC network contained the rated frequency predominantly with low harmonic content and flicker, the situation has changed with the increase in power electronic-based converters. Not only has this caused an increase in the harmonic content (in the total harmonic distortion, or THD [44]), switching transients has also increased. The critical case of switchgear, which is used as supplies for large electric vehicle charging stations, is an example where this phenomenon could become increasingly relevant. 

## 3. Technology: Sensors

The interest of operators of electrical equipment and machinery in condition monitoring and predictive maintenance is continuously increasing (cf. Section 1). The primary motivation is the avoidance of catastrophic failures, the reduction of operational cost, and the lifetime extension of the equipment. A key enabler for condition estimation and prognostic systems is sensor information capturing the relevant physical quantities. In the context of medium voltage switchgear, these are: (1) thermal status, (2) mechanical aspects of the switching and control equipment, as well as (3) partial discharge. 

### 3.1. Infrared Radiation Detectors for Remote Temperature Measurement

Thermal considerations are critical design criteria for electrical switchgear. Therefore, a continuous assessment of the thermal state is an important input to condition monitoring and prognosis systems. Several contacting temperature measurement techniques for electric equipment have been employed, based on surface acoustic wave sensors [45], RFID-sensors [46], or wireless sensors [47]. Contactless methods, such as infrared thermography (IRT) [48], have several advantages. Most importantly, the measurement does not interfere with the dielectric requirements of the equipment since it is non-contact [16,49,50,51] and free from electromagnetic interference [16,52] due to placement in regions of low magnetic or electric field. Furthermore, there is no need to shut down an energized system for inspection [16,53,54,55]. Moreover, IRT can cover a large area [16,51,54] unlike point measurement sensors. This drastically reduces the number of sensors needed.

Generally, there are three measuring principles for remote infrared temperature measurements: bolometric, pyroelectric, and thermoelectric. Pyroelectric sensors utilize the pyroelectric effect, which changes the spontaneous polarization in the pyroelectric crystal. Pyroelectric sensors are sensitive to changes in the IR scenery only. That means for constant measurement, modulation of the image on the sensor is required. Usually, this is realized by a “chopper wheel,” which covers the aperture of the sensor in a given frequency. Besides that, pyroelectric sensors show a high dependency on ambient temperature changes. Bolometers utilize the temperature dependency of electrical resistance, structured on a thin membrane. Incoming radiation heats the membrane and therefore changes the resistance. For high detectives, a high-temperature coefficient of the resistor material is needed, since the temperature changes of the membrane are relatively small. Therefore, ambient temperature changes will cause dramatic offset effects, if not compensated appropriately, as these temperature changes can be several magnitudes higher than the actual measurement signal caused by infrared radiation. Consequently, many microbolometers utilize a “shutter”, an element which can cover the optical path of the sensor and is used for an offset adjustment.

**Figure 8:**
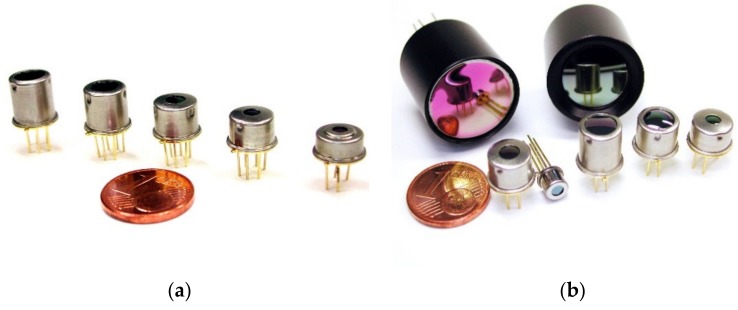
(**a**) Different optics and enclosures of the thermopile array sensors; (**b**) thermopile array sensors in different resolutions ranging from 8 × 8 to 120 × 84.

Thermoelectric sensors like thermopiles generate an output voltage proportional to the detected infrared radiation. In general, they consist of a series of thermocouples, structured on a thin membrane. The cold junctions of the thermocouples are structured on a heat sink to ensure a high-temperature gradient between the hot and cold junctions when the incoming infrared radiation changes the membrane temperature. Thermopiles (Figure 8a) are long time stable and do not require a mechanical movable element like a shutter or chopper. Besides that, the drift of sensitivity and offset is shallow, which makes them the ideal technology for long term monitoring and radiometric measurement.

All these sensor principles are available as a single point sensor or as arrays of several pixels, resulting in infrared images. Image sensors usually provide some monolithic integrated processing such as amplification, analog-digital-conversion units (ADCs), calibration data, and even sometimes image processing (Figure 8b).

To ensure a reliable, long-term stable high measurement accuracy over an extensive ambient temperature range, the thermopile technology was chosen in the FLEMING project.

### 3.2. Sensors for Breaker Drive Monitoring 

**Figure 9:**
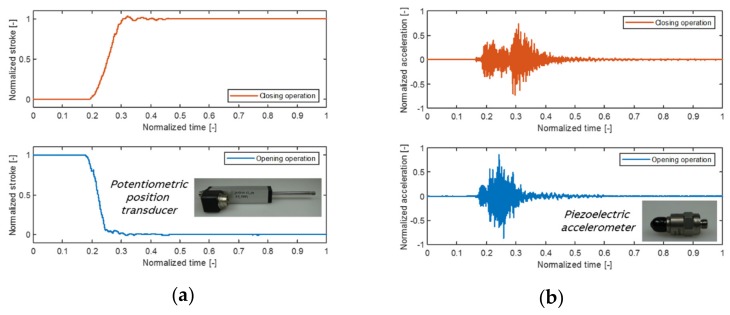
Monitoring options with possible sensor solutions: (**a**) Typical travel curve measured by a potentiometric position transducer; (**b**) Typical housing vibrations of a circuit breaker due to switching operations measured by a piezoelectric accelerometer. Source: Schematic measurements derived from own (ABB) tests of circuit breakers.

In the development of breaker drives, endurance tests are performed for the kinematic chain from the operating mechanism to the poles where the travel curve, speed, torsion, contact pressure, bouncing as well as vibrations are evaluated [56]. Therefore, the reliable and robust monitoring of breaker drives needs to be based on those quantities. The position of the moving contact and, accordingly, the travel curve is preferred to be measured directly by a linear transducer/potentiometer at the pushrod [33]. Figure 9a outlines a typical travel curve measurement for the closing and the opening operation of a circuit breaker. From the travel curve, the opening and closing speeds of the breaker drive are usually calculated. Alternatively, rotational transducers are used to derive the travel curve from the rotation of the main shaft, which only gives an estimation of opening and closing speeds [15]. The main characteristics of the breaker drive can be extracted from the travel curve for the development of a monitoring and diagnostics approach. In [57], new kinds of resistance strain force sensors are developed to measure the contact force in the operating mechanism of the circuit breaker. Furthermore, acceleration sensors offer the possibility of analyzing the vibrations of the circuit breaker [34,35]. Figure 9b shows representative vibration measurements at the circuit breaker housing for the closing and the opening operation. By performing signal processing methods and developing algorithms, the vibration signals can be used to detect mechanical anomalies of the breaker drive.

Further condition assessment methods and the developed sensing technology can be taken from [15], which provides a comprehensive but not exhaustive overview of relevant research work in the area of high voltage and medium voltage circuit breakers. To establish a robust and reliable monitoring and diagnostics system, sensors must be further developed to fit the requirements for measuring the main characteristics of a breaker drive.

### 3.3. Sensors for Partial Discharge Monitoring 

As discussed in Section 2.2, partial discharge (PD) measurements are among the main measurement techniques to assess the health of the electrical insulation in high voltage equipment. 

Several different measurement systems or approaches exist and are well-documented in literature and standards (cf., e.g., [58] and references cited therein, in particular [40,59,60].) [60] Capacitive, inductive, UHF/VHF, acoustic, and optical approaches are options to detect PD, often with the additional aim of identifying the short pulses corresponding to the discharges occurring at critical voltages or times. The main aim of such an analysis is to identify the defect concerning either its type, magnitude, or its origin. The localization of the origin of the defect is particularly crucial for large high or medium voltage equipment, to enable selective repair or replacement. 

A candidate for carrying out tests is an electro-magnetic, capacitive-coupling measurement system like the one given in [60]. Within the scope of this project, PD measurements will be carried out using both standard capacitive and inductive coupling methods together with high-end PD acquisition systems as well as the sensor mentioned above. Signal processing methods could then be used in the post-processing stage to evaluate PD activity. The sensor will then be benchmarked against the high-end PD acquisition systems.

## 4. Technology: Machine Learning for Predictive Maintenance

Artificial intelligence and autonomy are heavily discussed topics in politics, business networks, as well as industry associations and bodies [61]. In the industrial environment, AI seems to be following Industry 4.0 [62,63,64] as the next big hype. It is enhancing industrial systems from automation to production optimization to supply chain management [65,66]. Furthermore, industrial players are building on advancing autonomy in industrial systems through the application of AI methods [66,67,68].

In the previous section, different technologies have been shown, which can be used to monitor the condition of assets of the distribution grids and transfer planning information to a CMMS. These sensor data can then be used to make predictions about the health state of the system, or how much productive time is left until a failure occurs (RUL – remaining useful lifetime [69]). This information can then be used to schedule maintenance in advance of the predicted failure. In general, those techniques are used to avoid unplanned downtimes, which results in the more effective usage of resources.

Of course, there are alternatives to machine learning approaches, which have different advantages and disadvantages. Expert systems are based on a set of human-defined rules. They collect and conserve the knowledge of multiple experts and use it to make decisions. Simulation-based systems follow a similar approach. Simulations use the laws of physics and models of the system under observation to predict future states. The strength of both these approaches is their foundation in explainable principles that can be used to rationalize decisions. However, the cost of knowledge acquisition can be quite high. In our example, building adequate models for all types of switchgear is a Herculean task that few are willing or able to undertake.

Machine learning on the other hand has the advantage that it can (theoretically) work without any domain knowledge if enough data is available. With today’s technology, such data can be collected automatically at low cost. There is a major caveat, however, in that the complete data covering all states is rarely available. 

For these reasons, we see the use of data-driven approaches as a key enabler for industrially scalable systems. Still, human expertise can (and must) augment a machine learning approach and reduce the required amount of data through clever feature engineering. A non-expert would need to collect all data related to switchgear and would suffer heavily from the curse of dimensionality. Expert knowledge on the other hand can help to focus on the promising data sources and eliminate noise values. In the remainder of this section, we describe the principles of machine learning as used in our vision.

### 4.1. Data

In predictive maintenance, one usually deals with time-series data, which is collected from one (univariate) or multiple (multivariate) sensor(s) and contains dependencies in time. The training data can be collected from different settings, like reactive or preventive maintenance (Section 1). Using data collected in a reactive maintenance setting is an advantage for the learner, as the characteristics of failures can be found in the data. Therefore, precise predictions can be expected. If data from a preventive maintenance setting is used for machine learning, usually there is some healthy lifetime left at the point of maintenance, which will not be exploited. That is a disadvantage for machine learning, as no information about the failure could be collected, which makes precise predictions difficult. 

### 4.2. Preprocessing

An integral part of machine learning is to design a feature representation carrying information that can be exploited by a learning algorithm. One of the major problems of this part is the variability in length and measurement frequency of sensors often found in time series data, since many learning algorithms assume fixed-length feature vectors. An elegant way of tackling this problem is the use of tsfresh [70] which automatically constructs various features motivated by existing research and offers methods to choose from such a generated set. Alternatively, due to the recent rise of neural networks and deep learning, various attempts have been made to use architectures for creating fixed-length feature representations for time series data, for example, using an LSTM [71]. For more methods, we refer to [72].

Once such a fixed-length but potentially significant representation has been computed, one usually tries to limit the number of features to a reasonable amount to keep learning computationally tractable and to avoid the curse of dimensionality [73], while minimizing loss of relevant information concerning the original data. A common method to accomplish this is the Principal Component Analysis [74], creating new features as combinations of original ones, and only select those new features explaining most of the variance in the data.

Frequency data can be extracted by decomposing any signal into a sum of periodic components, sinusoidal functions, which can be used to transform the signal from the time domain to the frequency domain. The process of discovering the frequencies at which a signal oscillates by transforming it to the frequency domain is called the Fourier Transform. For discrete signals, the Fourier Transform is usually calculated using the Fast Fourier Transform (FFT) algorithm [75]. Similarly, the Power Spectral Density (PSD) describes the frequency spectrum of a signal. Besides, it also factors in the power distribution at each frequency bin, so that the surface below the frequency peaks correspond to the power distribution at each frequency [76]. Importantly, although FFTs have a very high resolution in the frequency domain, they do not provide any information about the time domain. In other words, the FFT tells us at which frequencies the signal oscillates, but not when the oscillations occur. Hence, performing an FFT is most suitable when the frequency spectrum is stationary as opposed to time-dependent. By contrast, a Wavelet Transformation has both frequency and temporal resolution. It is better suited for analyzing signals with a dynamic frequency spectrum, i.e., when the frequency spectrum changes over time. The Wavelet Transform uses functions that are localized in time that are convolved with the original signal.

IRT-based data also needs to be preprocessed with a series of algorithms. For example, IRT images are typically low-contrast. Since most object-recognition algorithms rely on color or brightness, measures to improve the contrast are often useful (see [77] or [78] as examples). Furthermore, especially at small resolutions, border pixels tend to be quite noisy. If an approach based on histograms, max/min values, and similar features is to be used, these pixels must be removed to avoid false conclusions.

### 4.3. Machine Learning for Predictive Maintenance

There are different typical targets for prediction, which will be described in the following. One target is to predict the health state of a system, e.g., good, bad, or worse, where the last state usually describes a faulty system. This prediction can also be used to estimate the remaining useful lifetime of a system. [79] use a support vector machine [80] to predict the probability distribution over a set of health states. Combined with the average RUL of the historical data of each state, a weighted sum of the average historical RULs for each health state is weighted by its associated probability, to receive a RUL prediction. [81] use a multiple binary classifier approach, where each classifier predicts healthy or faulty for a different prediction horizon. For each prediction horizon, the cost of maintenance is computed based on the probability of unplanned breaks and the probability of unexploited lifetime. The returned RUL equals the prediction horizon with the cheapest costs.

Another way is to predict the health index of the system, which describes the degradation of a system. [82] use a recurrent neural network (RNN) [83] to get a feature representation for the time series. This feature vector was used to train a k-nearest neighbor [84] algorithm. At prediction time, the RUL is estimated based on the weighted average of the RULs of the k most similar health index curves of the training process. 

There are also approaches to predict the RUL of the system directly. One approach is to first divide each instance into fixed-size non-overlapping windows, which are labeled with the corresponding RUL given by the instance. Based on these training instances, a support vector machine is trained. At prediction time, the given instance is also divided into the same fixed size windows, which overlap. For each of these instances, a support vector machine is used to predict the RUL. The returned value of this approach is the average of all RULs predicted for the windows of the given instance [85]. 

### 4.4. Artificial Intelligence Used in Switchgear Monitoring

Given the current popularity of AI, it is not surprising that there is already a large body of work addressing AI-based monitoring of electrical equipment. There are several IRT-based approaches to electrical equipment monitoring. For example, [86] trained an SVM with the Zernicke moments (i.e., polynomials that are orthogonal to the unit disk) of binarized IRT images in a substation [86]. [78] enhance IRT images of rotating machinery with nonsubsampled contourlet transform (NSCT) and feed a series of features taken from the image histogram to several machine learning algorithms such as SVM and feed-forward neural networks (NN) [78]. [87] test a series of features extracted from IRT images of electrical equipment with an SVM and a NN to classify faulty phases in switchgear [87]. A comprehensive overview of the advantages and disadvantages of various types of features taken from IRT images is given in [53]. However, all these approaches have in common that they require costly equipment that usually could not economically be installed in a switchgear.

Some work has also been done in the context of PD detection based on Artificial Intelligence algorithms. Among the methods used are K-means clustering [88,89], NN [90,91,92], and SVMs [90]. A relatively new approach uses a LSTM Recurrent Neural Network (RNN) and Ultra-High Frequency signals to diagnose PD [93]. Moreover, in [94], a boosting algorithm (i.e., RankBoost) is used to prioritizing maintenance of circuit breakers based on timing parameters, and in [95], rule-based algorithms are used to develop expert systems that output a composite risk index for circuit breakers based on monitoring parameters such as the age of the device and its history of failures. In [96], the authors incorporate known limits of circuit breaker monitoring values (e.g., number of operations, contact resistance, gas temperature) to develop fuzzy expert systems, as well as unsupervised learning algorithms (i.e., k-means and hierarchical clustering) to form clusters of data that correlate with the circuit breakers’ probability of failure. Finally, the same inputs were used to train a neural network that predicts the age of a circuit breaker. 

Based on the requirements of different stakeholders, we plan to develop an appropriate machine learning approach for predictive maintenance of medium voltage switchgear. Therefore, we would start with an analysis of the data to choose adequate preprocessing steps. Afterwards, we would want to compare different machine learning approaches. Each approach requires specific data preparation steps to prepare the data for the training process. Additionally, the parametrization of the preprocessing and the machine learning approach affect the performance. That is why we want to find a combination of preprocessing, machine learning approach, and parametrization that fits the given data.

## 5. Business Models

Building on power grids and sensors as smart infrastructure, the analysis of data with artificial intelligence, ultimately, must provide value to the stakeholders involved in manufacturing, using, and maintaining electrical switchgear. As an interdisciplinary research approach that combines engineering, information systems, and computer science, service science provides methods and tools with which networked business models, processes, and organizational structures can be designed and managed. The core property of ‘service’ is that value is co-created by stakeholders that cooperate in what is called a service system—a configuration of people, technologies, and other resources that interact with other service systems to create mutual value ([97], page 395). 

The service system we set out to design will enable stakeholders—including grid providers, manufacturing companies, and service providers—to improve the effectiveness and efficiency of maintaining switchgear in medium voltage energy grids. Further, is shall contribute to evolving the current supply grid into a smart grid [98]. This smart grid is expected to accommodate bidirectional energy flows since customers will get involved in energy generation, transmission, and consumption [99]. Also, the European Union’s vision of the smart grid is that it needs to be flexible, accessible, reliable, and economically sensible [5]. In case of an incident, some businesses using medium voltage switchgear will be unable to repair them because they lack expertise. Furthermore, repairing or replacing is more expensive and can account for power cuts, while foresighted maintenance makes resources plannable, thus ultimately improving lifespans of essential parts of the supply grid. Therefore, maintaining essential parts will become more crucial for a smart grid, putting it center-stage in our service system.

In our case, we want to minimize downtime while maximizing the lifespan of our equipment, thus, applying predictive maintenance (Sec 1). Predictive maintenance has been applied for different domains, e.g., agricultural [100], automotive [101] and industrial machinery [102]. The key to establishing predictive maintenance in the energy grid is the availability and analysis of appropriate data [103], enabled by the different sensors and monitoring techniques previously explained. With predictive maintenance, the quality of the supply grid may be improved. At the same time, repair costs can be minimized, failures can be reduced, and the longevity of essential components can be extended—assuming that sufficient amounts of data are at hand and algorithms identify data patterns that precede incidents with sufficient predictive accuracy. 

Apart from predictive maintenance, we consider current trends for our service system to enhance the value co-created by the stakeholders involved, e.g., the Internet of Things (IoT), and digital platforms, to recombine resources possessed by service systems [104]. The IoT refers to physical objects networked to the internet, enabling ubiquitous intelligence [105]. In our case, switchgear and other parts of the supply grid might be enabled to provide detailed condition data to an information system that predicts the failure of the components. Digital platforms can provide applications, shared commodities, social media, products, or digital services that extend the predictions. They can be multi-sided, mediating different stakeholders on the same technical core [106]. Digital platforms enable stakeholders to exchange information, goods, and services, which facilitates new business models [107,108]. In the case of our business model, a platform can be the medium on which switchgear manufacturers communicate with their customers. In a future scenario, customers might also rent switchgear from their providers, outsourcing maintenance processes in exchange for a time-/use-based fee instead of paying a fixed price to buy the switchgear. 

**Figure 10:**
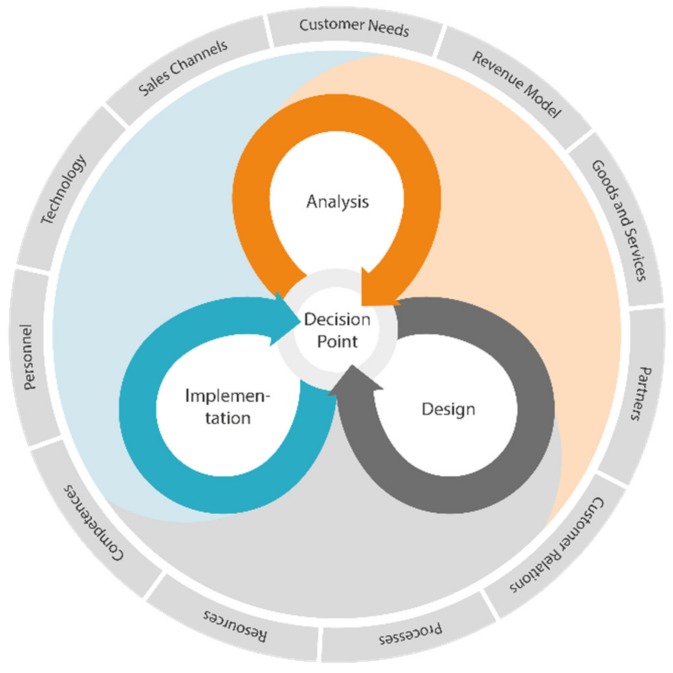
A reference process for designing smart service systems, translated from [109].

DIN SPEC 33,453 prescribes a nominal process for smart service systems engineering [109]. An overview of the process is presented in Figure 10. The reference process consists of three phases—analysis, design, and implementation—and specifies a series of activities and methods to instantiate these phases. The order in which to carry out the phases depends on the given context in which the service system is supposed to work, making the process flexible to instantiate. Also, designers can repeat a phase, e.g., if results obtained in any phase are insufficient. At the end of each phase, there is a decision point, at which users analyze the results of the phase and decide on how to proceed. This adds flexibility to the reference process and makes it applicable to a broader context by reacting to different requirements of the service system design. 

During the analysis phase, the customer requirements are analyzed to identify new ideas for digital services, prioritized, and tested for their feasibility and profitability. Activities in the analysis phase include market analysis, stakeholder analysis, and idea generation. The design phase aims to develop new services fulfilling the requirements analyzed previously. The digital service and the service system must be conceptualized, stakeholders and their roles must be defined, a prototype must be developed, and the prototype needs to be evaluated. The decision point of the design phase relies on the results of the evaluation. If it is enough, an implementation phase can follow. Otherwise, the design phase might be repeated. The implementation phase serves as a transition to manifest the designed service in the business. Activities include the planning of this transition, developing launch strategies, implementation of the service system, and lessons learned. In a ring around the reference process in Figure 10, there are the different design dimensions of a service system. They are subject to be defined and detailed as the process commences.

For developing a service system for realizing the mobility and energy transition, we start with an analysis phase to collect, structure, and prioritize the requirements of different stakeholders. We plan to continue with a design phase, designing and evaluating a prototype for the predictive maintenance of medium voltage switchgear. Next, we want to refine our service system with another analysis and design phase before transitioning to an implementation phase. We will adjust the process dynamically according to the results of each phase. After successfully applying this reference process, we will have designed a feasible and profitable business model for economic predictive maintenance of medium voltage switchgear, which builds on smart grid infrastructure and condition monitoring.

## 6. Limitations

This review paper presents an overview of the challenges and state-of-art for predictive maintenance for medium voltage switchgear and present a potential solution approach. We do not present a fully worked-out technical solution here, but instead provide detailed insights into the foundations and challenges for an economic ML-based predictive maintenance solution. Due to this nature, we might be missing technical challenges that only become visible when implementing the solutions.

The presented solution focusses on the use of machine learning for the predictive maintenance model development. Other solution approaches utilizing e.g., purely 1^st^ principle models, are not covered in detail. As the market is dominated by existing installations of customer-tailored medium voltage switchgear cabinets (“brownfield”), retrospective engineering of 1^st^ principle models does not economically scale, additionally to the technological challenges provided in Section 4. Similar solutions have been proposed for e.g., predictive maintenance of power substation equipment using infrared thermography and machine learning [110].

Apart from the presented fault-monitoring of switchgear, other sensing options could be introduced to detect and predict further faults [111]. For example, partial discharge may also be predicted by analyzing data from differential electric field sensors [112,113] or using the transient earth voltage method [114]. Overall, the selection of the right monitoring aspects strongly depends on the switchgear type and its intended application [111]. 

The paper assumes a limited scope for the monitoring systems. It explicitly excludes using the information in a SCADA system, or incorporating novel monitoring systems for multiple switchgear [115].

The described use-case and solution focus on a particular life-cycle phase of the switchgear i.e., maintenance. Concepts like digital twin and cyber-physical systems cover a broader range, or even the complete, life-cycle of assets. As predictive maintenance is the main use-case for industrial AI [11,12], focusing on this use-case initially seems to be reasonable, before broadening the scope to other life-cycle aspects like engineering or operation in future research. 

## 7. The Future Direction of Research

Besides the current state-of-the-art in switchgear monitoring described above and the potential developments of predictive maintenance for assets based on novel sensors and machine learning methods proposed above, further topics of open research remain.

### 7.1. Distribution Grid Assets and Monitoring

It is of interest if PD detection systems at an affordable price will find widespread acceptance in everyday operation. There seem to be good chances regarding the opportunities of digitalization and data analytics, that performance and usability levels can be reached, which generate convincing business cases. In real-world environments, still, separating the real PD from disturbances represent one of the biggest challenges in this kind of measurements [116]. This challenge is expected to increase with the higher penetration of power electronic devices. 

Condition monitoring and diagnostics of the breaker drive are a further topic of future research, as the kinematic chain is complex and consists of many mechanical parts, which may potentially fail [13].

The development of communication and sensor technology was strongly driven by the consumer electronics industry during the last decade. These technologies nowadays enter industrial applications and enable them to equip machinery with sensor networks [117]. The first examples of such sensor systems for usage in electrical installations have been demonstrated recently [118] and promise to pave the way to a digitalization of the electricity network. 

### 7.2. Machine Learning for Predictive Maintenance

Even though IRT condition monitoring has a long and successful tradition, there are still many open questions that need to be addressed to allow its widespread use. Unusual but harmless situations that can occur during grid operation need to be identified and understood. For example, a strong phase imbalance might appear as a fault even though it only reflects an unusual usage pattern. Machine learning algorithms must be trained in such a way that they correctly classify these cases as healthy.

The foundation of predictive maintenance is the collection of data via a variety of sensors. Each sensor can be of a different type, so that different sensor signals are collected. Each signal can be prepared for a machine learning approach via an appropriate preprocessing method, which results in a vast amount of parallel preprocessing steps for multiple sensors, where each of the preprocessing methods can have multiple hyperparameters. After preprocessing, the data can be used by a variety of machine learning approaches, suggesting maintenance, where each of the machine learning approaches can have multiple hyperparameters resulting in different performance. Manually creating pipelines consisting of preprocessing and learning algorithms, together with their hyper parametrization, is both a tedious and time-consuming task for data scientists and thus very costly. Accordingly, the field of automated machine learning (AutoML) is rapidly growing as it promises to automate this task partially. For other types of machine learning, there already exist a few AutoML tools, like ML-Plan [119] for multiclass classification, but one for predictive maintenance is still missing. This is an excellent opportunity for us to create such an AutoML tool and investigate challenges in the setting of predictive maintenance compared to the standard setting.

### 7.3. Requirements for the Adoption of AI Solutions in Industrial Practice

Adaptation of industrial AI solutions needs to be moderated, and AI providers need to address industry-specific requirements [120], like the introduction of Industrie 4.0 technologies [62,63,64]. Especially ethical implications need to be addressed, as they are currently heavily discussed in politics [121] and among industrial partners [122]. 

To develop a business model suitable for a CMMS based on predictive maintenance using artificial intelligence, we plan to apply a standard for service system engineering [109]. With this approach, we can assure to include the different requirements of all stakeholders included in the smart service system, e.g., network providers, municipal utilities, sensors manufacturers, and asset manufacturers. These requirements will be used to conceptualize a digital platform using the results of the machine learning algorithms for predictive maintenance in an economically viable way. 

The development process for data-driven services needs to be integrative and put the customer value into focus [123]. Furthermore, it should follow a structured process like CRISP-DM [124] with particular attention to process simplicity [125].

### 7.4. Security of Digital Systems

The renovation of the electricity distribution grid demands well-functioning information and communication technology to prevent system failures. To control and maintain the grid efficiently, grid operators must add interconnected components to their grids and establish an extensive networked CMMS. Due to the complexity of the emerging smart grid and its significant status for public order (critical infrastructure), experts expect higher risks to cyber-attacks, which include conventional attacks like DoS (Denial of Service) attacks, replay attacks, or false data injection. 

Furthermore, the skill level needed to attack industrial control systems is decreasing, as respective tools are becoming more available [126]. The surface for cyberattacks on grid infrastructure is also increasing, as traditional network protocols and commodity IT hardware are taking their place in the smart grid [126]. Potentially, approaches for analysis of anonymized or encrypted data, e.g., [127], may be evaluated to increase the security level for data leaving secured IT networks in the future.

Additionally, load frequency control devices, and new grid components that are connected to the internet are vulnerable to polluted input and output data that can corrupt the network performance enormously [128,129]. Currently, traditional IT protection techniques like VPNs, intrusion detection systems, and anti-virus software are used to protect the grid. However, the increasing interconnectivity between soft- and hardware creates a larger-scale cyber-physical system that potentially requires additional protection mechanisms compared to traditional protection measures [128].

## 8. Conclusions 

The electrical grid is currently undergoing significant changes, as energy production becomes more volatile through the increase in renewable energy sources and the increase in distributed demand sources, e.g., fast-charging stations for electric vehicles. Many activities have been initiated to address this challenge for the high voltage transmission grid, but few activities target the retrofitting of the medium voltage grid. 

With the present review paper, we show exemplarily how the existing medium voltage grid infrastructure can be adapted to the novel needs on an economic scale. Combining novel sensor technology and methods of machine learning may lead to predictive maintenance solutions for medium voltage switchgear, which are accompanied by an industry-fitting business model.

## References

[1] Schiffer H.-W. (2019). Zielvorgaben und staatliche Strategien für eine nachhaltige Energieversorgung. Wirtschaftsdienst.

[2] Renn O., Marshall J.P. (2016). Coal, nuclear and renewable energy policies in Germany: From the 1950s to the ‘Energiewende’. Energy Policy.

[3] (2019). Bericht zum Zustand und Ausbau der Verteilernetze 2018.

[4] Goop J., Odenberger M., Johnsson F. (2016). Distributed solar and wind power–Impact on distribution losses. Energy.

[5] European SmartGrids Technology Platform (2006). Vision and Strategy for Europe’s Electricity Networks of the Future. EUR 22040.

[6] Flexibilität im Stromversorgungssystem (2017). Bestandsaufnahme, Hemmnisse und Ansätze zur verbesserten Erschließung von Flexibilität.

[7] Schmautzer E., Lagler M.A. (2017). Neue Anforderungen an die Mittelspannungs- und Niederspannungs-Stromversorgung im städtischen und ländlichen Raum. Elektrotech. Inftech.

[8] Kumar G.V.B., Sarojini R.K., Palanisamy K., Padmanaban S., Holm-Nielsen J.B. (2019). Large Scale Renewable Energy Integration: Issues and Solutions. Energies.

[9] Mobley R.K. (2002). An Introduction to Predictive Maintenance.

[10] Friedman J., Hastie T., Tibshirani R. (2001). The elements of statistical learning.

[11] Rykov M. The Top 10 Industrial AI use cases. https://iot-analytics.com/the-top-10-industrial-ai-use-cases.

[12] Rykov M., Scully P. (2019). Industrial AI Market Report 2020–2025.

[13] Turrin S., Deck B., Egman M., Cavalli L. Medium voltage equipment monitoring and diagnostics: Technological maturity makes concepts compatible with expectations. Proceedings of the 23rd International Conference on Electricity Distribution.

[14] Labib A.W. (2004). A decision analysis model for maintenance policy selection using a CMMS. J. Qual. Maint. Eng..

[15] Uzelac N., Heinrich C., Pater R., Arnold J., Eichhoff D., Ferraro V., Gariboldi N., Germain M., Gioseffi A., Ito T. (2018). Non-intrusive methods for condition assessment of distribution and transmission switchgear. Technical Brochures 737.

[16] Jadin M.S., Taib S. (2012). Recent progress in diagnosing the reliability of electrical equipment by using infrared thermography. Infrared Phys. Technol..

[17] Russell S., Norvig P. (2002). Artificial Intelligence: A Modern Approach.

[18] Amihai I., Gitzel R., Kotriwala A.M., Pareschi D., Subbiah S., Sosale G. An Industrial Case Study Using Vibration Data and Machine Learning to Predict Asset Health. Proceedings of the 2018 IEEE 20th Conference on Business Informatics (CBI).

[19] Gitzel R., Amihai I., Garcia Perez M. Towards Robust ML-Algorithms for the Condition Monitoring of Switchgear. Proceedings of the 1st Conference on Societal Automation.

[20] Amihai I., Chioua M., Gitzel R., Kotriwala A.M., Pareschi D., Sosale G., Subbiah S. Modeling Machine Health Using Gated Recurrent Units with Entity Embeddings and K-Means Clustering. Proceedings of the 2018 IEEE 16th International Conference on Industrial Informatics (INDIN).

[21] Shahidehpour M., Ferrero R. (2005). Time management for assets: Chronological strategies for power system asset management. IEEE Power Energy Mag..

[22] Lee J., Bagheri B., Kao H.-A. (2015). A cyber-physical systems architecture for industry 4.0-based manufacturing systems. Manuf. Lett..

[23] Beverungen D., Müller O., Matzner M., Mendling J., vom Brocke J. (2019). Conceptualizing smart service systems. Electron. Mark..

[24] Uhlemann T.H.-J., Lehmann C., Steinhilper R. (2017). The digital twin: Realizing the cyber-physical production system for industry 4.0. Procedia Cirp.

[25] Tao F., Qi Q., Wang L., Nee A.Y.C. (2019). Digital Twins and Cyber–Physical Systems toward Smart Manufacturing and Industry 4.0: Correlation and Comparison. Engineering.

[26] Qi Q., Tao F. (2018). Digital Twin and Big Data Towards Smart Manufacturing and Industry 4.0: 360 Degree Comparison. IEEE Access.

[27] Zhang X., Gockenbach E., Wasserberg V., Borsi H. (2007). Estimation of the Lifetime of the Electrical Components in Distribution Networks. IEEE Trans. Power Delivery.

[28] Zhang X., Gockenbach E. (2007). Component reliability modeling of distribution systems based on the evaluation of failure statistics. IEEE Trans. Dielectr. Electr. Insul..

[29] (1996). IEEE Guide for Diagnostics and Failure Investigation of Power Circuit Breakers. IEEE Std C37.10-1995.

[30] (2001). IEEE Guide for the Selection of Monitoring for Circuit Breakers. IEEE Std C37.10.1-2000.

[31] Paoletti G.J., Herman G. Monitoring of electrical equipment failure indicators and zero-planned outages: Past, present and future maintenance practices. Proceedings of the Industry Applications Society 60th Annual Petroleum and Chemical Industry Conference.

[32] Saeli C., Serpellini F., Gatti C., Bianco A., de Natale G. How to guarantee continuity of supply and reliability in the smart grids?. Diagnostic systems in the MV network components, circuit breakers and switch disconnectors. In Proceedings of the 2012 Petroleum and Chemical Industry Conference Europe Conference Proceedings (PCIC EUROPE).

[33] Andruşcă M., Adam M., Pantelimon R., Baraboi A. About diagnosis of circuit breakers. Proceedings of the 2013 8th International Symposium on Advanced Topics in Electrical Engineering (ATEE).

[34] Lai M.L., Park S.Y., Lin C.C., Naidu H., Soom A., Reinhorn A.M., Lee Y.H., Soong T.T., Demjanenko V., Benenson D.M. (1988). Mechanical failure detection of circuit breakers. IEEE Trans. Power Delivery.

[35] Hoidalen H.K., Runde M. (2005). Continuous monitoring of circuit breakers using vibration analysis. IEEE Trans. Power Delivery.

[36] Lee D.S.S., Lithgow B.J., Morrison R.E. (2003). New fault diagnosis of circuit breakers. IEEE Trans. Power Delivery.

[37] Hou N. The infrared thermography diagnostic technique of high-voltage electrical equipments with internal faults. Proceedings of the POWERCON ’98. 1998 International Conference on Power System Technology. Proceedings (Cat. No.98EX151).

[38] Craig T. Condition monitoring in low voltage circuit breaker technology. Proceedings of the IET International Conference on Resilience of Transmission and Distribution Networks (RTDN 2017).

[39] Huda A.S.N., Taib S. (2013). Application of infrared thermography for predictive/preventive maintenance of thermal defect in electrical equipment. Appl. Therm. Eng..

[40] IEC (2000). IEC 60270, High-voltage test techniques—Partial discharge measurement, Version 2000.

[41] Janus P. (2012). Acoustic Emission Properties of Partial Discharges in the Time-Domain and Their Applications.

[42] Küchler A. (2017). Hochspannungstechnik (in german), esp. Ch 3.6: Teilentladungen.

[43] Florkowski M., Florkowska B., Zydron P. Influence of high voltage harmonics on partial discharge patterns modulation. Proceedings of the 2014 ICHVE International Conference on High Voltage Engineering and Application.

[44] (2014). IEEE Recommended Practice and Requirements for Harmonic Control in Electric Power Systems. IEEE Std 519-2014 (Revision of IEEE Std 519-1992).

[45] Stroganov K., Kronidov T., Luylin B., Kalinin V., Plessky V.P. SAW temperature sensors for electric power transmission lines. Proceedings of the European Frequency and Time Forum (EFTF).

[46] Wang B., Law M.-K., Yi J., Tsui C.-Y., Bermak A. (2019). A -12.3 dBm UHF Passive RFID Sense Tag for Grid Thermal Monitoring. IEEE Trans. Ind. Electron..

[47] Lu H., Yuan Y. Substation equipment temperature monitoring system design based on self-powered wireless temperature sensors. Proceedings of the 2nd International Conference on Systems and Informatics (ICSAI).

[48] Wildermuth S., Ahrend U., Hochlehnert M. Infrared Temperature Measurement System for Condition Monitoring of High Voltage Generator Circuit Breakers. Proceedings of the 17. ITG/GMA Symposium, Sensors and Measuring Systems.

[49] Glowacz A., Glowacz Z. (2017). Diagnosis of the three-phase induction motor using thermal imaging. Infrared Phys. Technol..

[50] Bagavathiappan S., Lahiri B.B., Saravanan T., Philip J., Jayakumar T. (2013). Infrared thermography for condition monitoring—A review. Infrared Phys. Technol..

[51] Chaturvedi D.K., Iqbal M.S., Pratap M. Intelligent health monitoring system for three phase induction motor using infrared thermal image. Proceedings of the 2015 international conference on energy economics and environment (ICEEE).

[52] Li B., Zhu X., Zhao S., Niu W. HV Power Equipment Diagnosis Based on Infrared Imaging Analyzing. Proceedings of the 2006 International Conference on Power System Technology.

[53] Smedberg M. (2006). Thermographic decision support--detecting and classifying faults in infrared images. Master’s Thesis.

[54] Chou Y., Yao L. Automatic Diagnostic System of Electrical Equipment Using Infrared Thermography. Proceedings of the 2009 International Conference of Soft Computing and Pattern Recognition.

[55] So A.T.P., Chan W.L., Tse C.T., Lee K.K. Fuzzy logic based automatic diagnosis of power apparatus by infrared imaging. Proceedings of the Second International Forum on Applications of Neural Networks to Power Systems.

[56] Perdon K., Scarpellini M., Magoni S., Cavalli L. (2017). Modular online monitoring system to allow condition-based maintenance for medium voltage switchgear. CIRED.

[57] Tang J., Lu S., Xie J., Cheng Z. (2017). Contact Force Monitoring and Its Application in Vacuum Circuit Breakers. IEEE Trans. Power Delivery.

[58] Hauschild W., Lemke E. (2019). High-Voltage Test and Measuring Techniques, Chapter 4: Partial Discharge Measurement.

[59] IEC (2016). IEC TS 62478: High voltage test techniques—Measurement of partial discharges by electromagnetic and acoustic methods.

[60] ABB Ability(TM) Condition Monitoring for switchgear – SWICOM. https://new.abb.com/medium-voltage/service/advanced-services/condition-monitoring-for-switchgear-SWICOM.

[61] Franke U., Sartori P. (2019). Machine politics: Europe and the AI revolution.

[62] Drath R., Horch A. (2014). Industrie 4.0: Hit or hype? [Industry Forum]. IEEE Ind. Electron. Mag..

[63] Krueger M., Drath R., Koziolek H., Ouertani Z. (2014). A new era. ABB Rev..

[64] Drath R., Matthias B., Horch A., Krüger M., Listmann K., Forschungszentrum A.B.B., Sendler U. Das Internet der Dinge, Dienste und Menschen. Industrie 4.0 grenzenlos.

[65] Zhang X., Ming X., Liu Z., Yin D., Chen Z., Chang Y. (2019). A reference framework and overall planning of industrial artificial intelligence (I-AI) for new application scenarios. Int. J. Adv. Manuf. Technol..

[66] Ahlborn K., Bachmann G., Biegel F., Bienert J., Falk S., Fay A., Gamer T., Garrels K., Grotepass J., Heindl (2019). Plattform Industrie 4.0: Technology Scenario ‘Artificial Intelligence in Industrie 4.0′.

[67] Gamer T., Kloepper B., Hoernicke M. The way toward autonomy in industry-taxonomy, process framework, enablers, and implications. Proceedings of the IECON 2019-45th Annual Conference of the IEEE Industrial Electronics Society.

[68] Gamer T., Hoernicke M., Kloepper B., Bauer R., Isaksson A.J. (2019). The Autonomous Industrial Plant-Future of Process Engineering, Operations and Maintenance. IFAC-PapersOnLine.

[69] Banjevic D. (2009). Remaining useful life in theory and practice. Metrika.

[70] Christ M., Braun N., Neuffer J., Kempa-Liehr A.W. (2018). Time series feature extraction on basis of scalable hypothesis tests (tsfresh—a python package). Neurocomputing.

[71] Hochreiter S., Schmidhuber J. (1997). Long short-term memory. Neural Comput..

[72] Bagnall A., Lines J., Bostrom A., Large J., Keogh E. (2017). The Great Time Series Classification Bake Off: A Review and Experimental Evaluation of Recent Algorithmic Advances. Data Min. Knowl. Discov..

[73] Marimont R., Shapiro M. (1979). Nearest neighbour searches and the curse of dimensionality. IMA J. Appl. Math..

[74] Wold S., Esbensen K., Geladi P. (1987). Principal component analysis. Chemom. Intell. Lab. Syst..

[75] Duhamel P., Vetterli M. (1990). Fast Fourier transforms: A tutorial review and a state of the art. Signal Process..

[76] Stoica P., Moses R.L. (2005). Spectral Analysis of Signals.

[77] Ryuichi I. (2002). New detection method of faulty distribution power apparatus using thermal images. SPIE.

[78] Bai T., Zhang L., Duan L., Wang J. (2016). NSCT-based infrared image enhancement method for rotating machinery fault diagnosis. IEEE Trans. Instrum. Meas..

[79] Kim H.-E., Tan A.C.C., Mathew J., Choi B.-K. (2012). Bearing fault prognosis based on health state probability estimation. Expert Syst. Appl..

[80] Drucker H., Burges C.J.C., Kaufman L., Smola A.J., Vapnik V. (1998). Support vector regression machines. Advances in neural Information Processing Systems.

[81] Susto G.A., Schirru A., Pampuri S., McLoone S., Beghi A. (2014). Machine learning for predictive maintenance: A multiple classifier approach. IEEE Trans. Ind. Inf..

[82] Gugulothu N., TV V., Malhotra P., Vig L., Agarwal P., Shroff G. (2017). Predicting remaining useful life using time series embeddings based on recurrent neural networks. arXiv.

[83] Rumelhart D.E., Hinton G.E., Williams R.J. (1986). Learning representations by back-propagating errors. Nature.

[84] Altman N.S. (1992). An introduction to kernel and nearest-neighbor nonparametric regression. Am. Stat..

[85] Khelif R., Chebel-Morello B., Malinowski S., Laajili E., Fnaiech F., Zerhouni N. (2016). Direct remaining useful life estimation based on support vector regression. IEEE Trans. Ind. Electron..

[86] Rahmani A.J., Haddadnia O. Seryasat Intelligent fault detection of electrical equipment in ground substations using thermo vision technique. Proceedings of the 2010 2nd International Conference on Mechanical and Electronics Engineering.

[87] Jadin M.S., Taib S., Ghazali K.H. (2014). Feature extraction and classification for detecting the thermal faults in electrical installations. Measurement.

[88] Peng X., Zhou C., Hepburn D.M., Judd M.D., Siew W.H. (2013). Application of K-Means method to pattern recognition in on-line cable partial discharge monitoring. IEEE Trans. Dielectr. Electr. Insul..

[89] Lin Y.-H. (2011). Using k-means clustering and parameter weighting for partial-discharge noise suppression. IEEE Trans. Power Delivery.

[90] Li L., Tang J., Liu Y. (2015). Partial discharge recognition in gas insulated switchgear based on multi-information fusion. IEEE Trans. Dielectr. Electr. Insul..

[91] Chang C.S., Jin J., Chang C., Hoshino T., Hanai M., Kobayashi N. (2005). Separation of corona using wavelet packet transform and neural network for detection of partial discharge in gas-insulated substations. IEEE Trans. Power Delivery.

[92] Si W.R., Li J.H., Li D.J., Yang J.G., Li Y.M. (2010). Investigation of a comprehensive identification method used in acoustic detection system for GIS. IEEE Trans. Dielectr. Electr. Insul..

[93] Nguyen M.-T., Nguyen V.-H., Yun S.-J., Kim Y.-H. (2018). Recurrent neural network for partial discharge diagnosis in gas-insulated switchgear. Energies.

[94] Zhong J., Li W., Wang C.Y.J. (2018). A RankBoost-based data-driven method to determine maintenance priority of circuit breakers. IEEE Trans. Power Delivery.

[95] Vianna E., Abaide A., Canha L., Miranda V. (2017). Substations SF6 circuit breakers: Reliability evaluation based on equipment condition. Electr. Power Syst. Res..

[96] Zarkovic M., Stojkovic Z. (2019). Artificial intelligence SF6 circuit breaker health assessment. Electr. Power Syst. Res..

[97] Maglio P., Vargo S., Caswell N., Spohrer J. (2008). The Service System Is the Basic Abstraction of Service Science. Inf. Syst. e-Bus. Manag..

[98] (2009). Report to NIST on the Smart Grid Interoperability Standards Roadmap.

[99] Gergen M.J., Campopiano M.T., Meyer A.H. (2014). CPUC Opens Rulemaking to Incorporate Distributed Energy Resources Into Grid Planning Process for California’s Investor-Owned Utilities.

[100] Lüttenberg H., Bartelheimer C., Beverungen D. Designing Predictive Maintenance for Agricultural Machines. Proceedings of the Proceedings of the Twenty-Sixth European Conference on Information Systems (ECIS2018).

[101] Dhall R., Solanki V. (2017). An IoT Based Predictive Connected Car Maintenance. Int. J. Interact. Multimed. Artif. Intell..

[102] Yan Y., Qi D., Gu H. (2017). A Real-Time IR-Fusion Switchgear Contact Monitoring System (SCMS). IEEE Access.

[103] Rudin C., Waltz D., Anderson R.N., Boulanger A., Salleb-Aouissi A., Chow M., Dutta H., Gross P.N., Huang B., Ierome S. (2012). Machine learning for the New York City power grid. IEEE Trans. Pattern Anal. Mach. Intell..

[104] Beverungen D., Lüttenberg H., Wolf V. (2018). Recombinant service systems engineering. Bus. Inf. Syst. Eng..

[105] Xia F., Yang L.T., Wang L., Vinel A. (2012). Internet of things. Int. J. Commun. Syst..

[106] Boudreau K.J., Hagiu A. (2009). Platform rules: Multi-sided platforms as regulators. Platforms, Mark. Innov..

[107] Eisenmann T., Parker G., van Alstyne M.W. (2006). Strategies for two-sided markets. Harv. Bus. Rev..

[108] Parker G., van Alstyne M. (2012). A Digital Postal Platform: Definitions and a Roadmap.

[109] DIN SPEC 33453— (2019). Entwicklung digitaler Dienstleistungssysteme; Norm, Deutsches Institut für.

[110] Ullah I., Yang F., Khan R., Liu L., Yang H., Gao B., Sun K. (2017). Predictive maintenance of power substation equipment by infrared thermography using a machine-learning approach. Energies.

[111] Biasse J.-M., Ferraro V., Brun P., Yang Y., Wang G. New features for MV switchgear are now available to move to condition based maintenance. Proceedings of the 2016 International Conference on Condition Monitoring and Diagnosis (CMD).

[112] Hussain G.A., Hummes D., Shafiq M., Safdar M.S. Proceedings of the 2019 IEEE Texas Power and Energy Conference (TPEC).

[113] Hussain G.A., Shafiq M., Lehtonen M., Hashmi M. (2015). Online Condition Monitoring of MV Switchgear Using $ D $-Dot Sensor to Predict Arc-Faults. IEEE Sens. J..

[114] Zhang C., Dong M., Ren M., Huang W., Zhou J., Gao X., Albarracín R. (2018). Partial discharge monitoring on metal-enclosed switchgear with distributed non-contact sensors. Sensors.

[115] Hou Z., Wu J., Ren S., Yang C., Mao C., Li H. Development of a Novel Comprehensive Online Monitor for MV Switchgears Based on Modbus. Proceedings of the 2018 8th International Conference on Power and Energy Systems (ICPES).

[116] Romano P., Parastar A., Imburgia A., Blennow J., Bongiorno M., di Tommaso A.O., Hammarström T., Serdyuk Y. Partial Discharge Measurements under DC Voltages Containing Harmonics Produced by Power Electronic Devices. Proceedings of the 2018 IEEE Conference on Electrical Insulation and Dielectric Phenomena (CEIDP).

[117] Ahrend U., Aleksy M., Berning M., Gebhardt J., Mendoza F., Schulz D. Challenges of the digital transformation: The role of sensors, sensor networks, IoT-devices, and 5G. Proceedings of the 2019 First International Conference on Societal Automation (SA).

[118] Laitinen T., Lyly T., Stenstrand M., Tammi J., Albrecht R., Nyberg J., Saksela K. Wireless sensor units for acoustic monitoring of switching devices. Proceedings of the CIGRE Session; Paris.

[119] Mohr F., Wever M., Hüllermeier E. (2018). ML-Plan: Automated machine learning via hierarchical planning. Mach. Learn..

[120] Hoffmann M.W., Drath R., Ganz C. Proposal for requirements on industrial AI solutions. Proceedings of the ML4CPS 2020.

[121] (2019). High-Level Expert Group on Artificial Intelligence (HLEG AI), Ethics guidelines for trustworthy AI.

[122] (2020). ZVEI Guidelines of the electrical industry for the responsible use of data and platforms.

[123] Krueger M., Chew E.K., Ouertani Z., Gitzel R. Integrative Service Innovation: An Industrial Use Case. Proceedings of the IEEE 17th International Conference on Business Informatics.

[124] Wirth R., Hipp J. CRISP-DM: Towards a standard process model for data mining. Proceedings of the Proceedings of the 4th international conference on the practical applications of knowledge discovery and data mining.

[125] Kloepper B., Hoffmann M.W., Ottewill J.R. (2020). Stepping up value in AI industrial projects with co-innovation. ABB Review.

[126] (2019). Critical Infrastructure Protection- Actions Needed to Address Significant Cybersecurity Risks Facing the Electric Grid.

[127] Marrella A., Monreale A., Kloepper B., Krueger M.W. Privacy-Preserving Outsourcing of Pattern Mining of Event-Log Data-A Use-Case from Process Industry. Proceedings of the 2016 IEEE International Conference on Cloud Computing Technology and Science (CloudCom).

[128] Stoustrup J., Annaswamy A., Chakrabortty A., Qu Z. (2019). Smart Grid Control: Overview and Research Opportunities.

[129] Peng C., Sun H., Yang M., Wang Y.-L. (2019). A Survey on Security Communication and Control for Smart Grids Under Malicious Cyber Attacks. IEEE Trans. Syst. Man, Cybern. Syst..

